# “Lost in Transition”: Informational Needs of Sepsis Survivors and Their Relatives Across the Care Trajectory—A Qualitative Study

**DOI:** 10.3390/jcm15010091

**Published:** 2025-12-23

**Authors:** Frank Vahl, Susanne Ullmann, Lea Draeger, Lena Kannengießer, Mathias W. Pletz, Claudia T. Matthaeus-Kraemer, Carolin Fleischmann-Struzek

**Affiliations:** 1 Institute of Infectious Diseases and Infection Control, Jena University Hospital, 07740 Jena, Germany; 2 Center for Sepsis Control and Care, Jena University Hospital, 07740 Jena, Germany; 3 Sepsis Foundation, c/o Department of Anesthesiology and Intensive Care Medicine, Charité—Universitätsmedizin Berlin, 12203 Berlin, Germany; 4 Institute of General Practice and Family Medicine, Jena University Hospital, 07740 Jena, Germany; 5 Institute of Social Medicine and Health Systems Research, Otto-von-Guericke University, 39106 Magdeburg, Germany

**Keywords:** sepsis, informational needs, sepsis survivors, discharge planning, rehabilitation, post-sepsis care, health communication, follow-up care, critical care

## Abstract

**Background/Objectives:** Sepsis survivors frequently experience long-term complications known as Post-Sepsis Syndrome. Many survivors and their relatives express ongoing dissatisfaction with the quality and accessibility of health information. Yet the specific informational needs and preferred formats remain insufficiently defined. To identify the informational needs of sepsis survivors and their relatives across different stages of illness and recovery. **Methods:** This qualitative study, conducted within the AVENIR project, included semi-structured telephone interviews with 12 survivors and 6 relatives in Germany. Interviews were transcribed verbatim and analyzed using qualitative content analysis according to Mayring. **Results:** Eighteen interviews highlighted phase-specific gaps in information. Relatives reported urgent needs for timely, comprehensible and empathetic communication during the ICU phase, often while under decision pressure. Survivors described limited capacity to process information during the acute phase and sought orientation only after cognitive and emotional stabilization. After discharge, both groups reported an “information vacuum”, marked by insufficient guidance on long-term physical and psychological consequences, rehabilitation, vaccination, and follow-up care. Many participants received no informational material, or only general or inconsistent information. Desired content emphasized basic sepsis knowledge, explanations of persistent symptoms, practical coping strategies, and navigation of support services. Preferred formats included peer support and repeated, personal conversations with healthcare professionals, complemented by trusted online and printed resources. **Conclusions:** Sepsis survivors and relatives experience notable, role- and phase-specific information deficits that extend from the ICU into long-term recovery. Timely, reliable, and accessible information may help reduce uncertainty, support coping, and strengthen autonomy for both survivors and relatives.

## 1. Introduction

Sepsis is a life-threatening condition caused by a dysregulated host response to infection. It remains a major global contributor to morbidity and mortality. According to estimates from the Global Burden of Disease Study, approximately 49 million people develop sepsis each year, and around 11 million die—representing nearly one in five deaths worldwide [1]. In Germany, sepsis also poses a substantial challenge to the healthcare system. An analysis of national hospital discharge data reported an incidence of 158 per 100,000 inhabitants, with an in-hospital mortality of 41.7% among identified cases. However, due to the low sensitivity of health claims data, true incidence is likely underestimated and mortality overestimated [2].

At the same time, improved intensive care has increased survival, but many survivors experience persistent impairments. A significant proportion develop Post-Sepsis Syndrome (PSS), which includes cognitive dysfunction, physical weakness, fatigue, anxiety, depression, and a high risk of newly acquired dependency on long-term care [3,4,5]. These sequelae overlap with symptoms of Post-Intensive Care Syndrome (PICS) and may last for months or years [6,7,8].

Despite the growing evidence on long-term consequences, structured aftercare remains insufficient in many settings [9,10]. Studies show that sepsis survivors often leave the hospital without clear follow-up plans, rehabilitative referrals, or targeted information about possible long-term effects [11,12,13]. Relatives also play a central role. They frequently take on informal caregiving responsibilities yet are rarely integrated into communication processes. Many relatives report emotional strain, uncertainty and a lack of guidance during and after hospitalization [14].

Timely and relevant information can help patients and families cope with the illness and its consequences. However, informational needs change across the disease trajectory, from the acute treatment phase to long-term recovery. Several studies document substantial sepsis-specific information deficits [15,16,17]. Many affected individuals are unfamiliar with what sepsis is, how it develops, and which long-term consequences may arise [18]. Sepsis-related topics are often missing from available materials, and many survivors receive no written information after discharge. Despite these recurring observations, there has been no systematic investigation into which specific information is lacking, when it is most urgently needed, and how it should be effectively tailored to survivors and relatives. This gap continues to limit the development of patient-centered communication strategies.

Qualitative research is well suited to identify these unmet needs because it provides deep insight into lived experiences of care. Building on this approach, the present study examines the informational needs of sepsis survivors and their relatives across different stages of illness and recovery. The study aims to identify shortcomings in current communication practices and to derive recommendations for needs-oriented information tools. It focuses not only on content and timing of information but also on preferred communication formats.

This work further expands the literature by mapping informational needs across multiple phases of the sepsis trajectory and by distinguishing the perspectives of survivors and relatives. By describing how readiness to receive and process information changes over time and differs between roles, it provides phase- and role-specific insights that may support more tailored communication strategies.

## 2. Materials and Methods

### 2.1. Background of the Study

This exploratory study is part of the AVENIR project (FKZ 01VSF21031), which aims to improve understanding of medical care and subjective experiences before, during, and after sepsis [19]. The project seeks to inform recommendations for organizing follow-up care and developing patient-centered informational materials, with active involvement of affected individuals. The study was pre-registered (DRKS00031302) and approved by the Jena University Hospital institutional review board (2023-2992-Daten).

### 2.2. Participants

Participants were recruited nationwide through social media advertisements, the sepsis foundation’s advisory network and flyers distributed in the post-intensive care unit of the Charité University Medicine Berlin. Although one recruitment pathway involved a single university hospital, participants themselves came from different regions across Germany. Eligibility criteria were age 18 years or older, fluency in German, and a sepsis diagnosis within the past five years. Both sepsis survivors and relatives who voluntarily agreed to participate were included. Recruitment continued until conceptual saturation was reached (see Section 2.5 and Section 3.1).

### 2.3. Data Acquisition

Data were collected through semi-structured telephone interviews with sepsis survivors and/or their relatives between May and November 2023. The interviews explored participants’ experiences with receiving a sepsis diagnosis and the communication of and supply with relevant medical information throughout the course of illness and recovery.

### 2.4. Expert Interviews: Characteristics and Setting

A semi-structured interview guide was used to explore core themes including general understanding of sepsis, experiences during hospitalization and communication with healthcare professionals, discharge and transitional care, rehabilitation and follow-up, initial diagnosis and communication, knowledge of sepsis before and after illness, psychological and social consequences, prevention and relapse, and moments of greatest informational need (Table 1).

All interviews were conducted via telephone by a trained final-year medical student (FV), under the supervision of an experienced social scientist (CTMK, M.A. in Educational Science, Psychology, and Sociology). CTMK has extensive expertise in conducting and analyzing qualitative interviews in healthcare and quality improvement settings. There was no prior relationship between interviewer and participants. Interviewees were informed in advance about the interviewer’s background and role. The interviews were conducted by telephone in a private setting with only the interviewer, the participant, and—in a few cases—accompanying relatives present.

### 2.5. Expert Interviews: Data Analysis

#### 2.5.1. Analytical Approach

All interviews were audio-recorded and transcribed verbatim. Analysis followed qualitative content analysis, a systematic and rule-governed method of text interpretation [20]. The analytical process comprised three steps:

1. Open coding of relevant text segments,
2. Development of thematic categories, and
3. Abstraction into overarching core categories.

#### 2.5.2. Assessment of Saturation

We monitored saturation throughout the analysis by assessing whether additional interviews contributed new conceptual insights. Because the study aimed to understand informational needs across the full sepsis trajectory, we assessed saturation for the dataset as a whole rather than for survivors and relatives separately. We applied the concept of conceptual saturation, meaning that no new themes, meaning units, or dimensions emerged. After saturation was reached, three confirmatory interviews were conducted to test the stability of the category system. These interviews did not add new concepts, and recruitment was therefore concluded.

#### 2.5.3. Reflexivity and Coding Validation

Reflexivity was maintained throughout data collection and analysis. The interviewer (FV) and the supervising social scientist (CTMK) engaged in regular discussions about potential influences of their professional backgrounds, expectations, or emotional engagement. Coding reliability was strengthened through iterative review: the interviewer conducted initial coding, and coding decisions were then refined through discussion until consensus was reached. As is standard in qualitative research, no statistical interrater metrics were calculated.

#### 2.5.4. Data Management

Data were managed and analyzed using MAXQDA (version 24; VERBI Software, Berlin, Germany). Definitions of categories and selected original quotes are provided in Electronic Supplement S1. The study methods are reported in accordance with the Consolidated Criteria for Reporting Qualitative Research (COREQ) checklist [21].

## 3. Results

### 3.1. Interview and Participant Characteristics

In total, 18 interviews were included (12 survivors, 6 relatives). The median interview duration was 37 min, resulting in a total interview time of approximately 11 h and 15 min. Conceptual saturation was reached after 15 interviews. We then conducted three additional interviews to confirm the stability of the category system; these interviews did not introduce new concepts.

The median age of survivors and relatives was 53 years. All 12 survivors had received intensive care unit (ICU) treatment, and 10 had participated in post-ICU rehabilitation. The six relatives described providing essential emotional and practical support during hospitalization and recovery.

### 3.2. Diagnoses Communication

Five survivors said they were informed about the diagnosis by a physician, four by relatives, and two through their own review of medical records. Among relatives, five of six learned about the sepsis diagnosis from a physician. Participants also reported considerable variation in when and where they received this information. Three relatives and seven survivors were informed during the ICU stay, two participants received the information before admission, and six could not recall the timing. Among survivors, eight learned about the diagnosis in the ICU, one before admission, and three could not remember. Among relatives, two were informed in the ICU, one before admission, and three could not recall.

**Figure 1:**
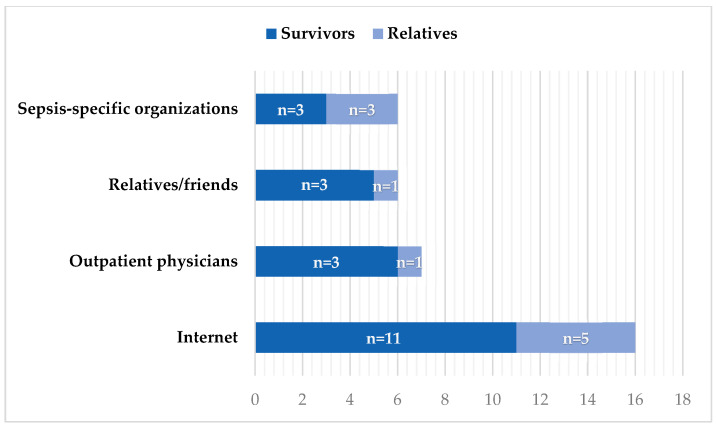
Next information sources following the diagnosis (*n* = 18; Survivors *n* = 12; Relatives *n* = 6).

After learning about the diagnosis, most participants sought additional information on their own. Figure 1 shows the main information sources used. Experiences with online information varied widely: some described feeling overwhelmed (“There’s a lot on the internet, but it’s overwhelming and you don’t know what really applies to you.”), while others highlighted the value of online forums: “What helped me most was a forum. That’s where I got concrete answers.”

### 3.3. Timing of Informational Needs

**Figure 2:**
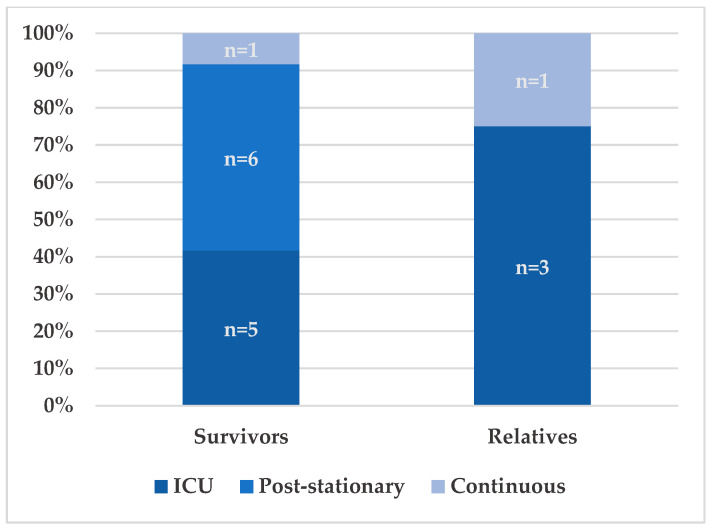
Timing of informational needs during and after hospitalization (*n* = 18; Survivors *n* = 12; Relatives *n* = 6).

Informational needs changed over time and differed between survivors and relatives. Figure 2 illustrates when these needs arose during and after hospitalization. A strong demand for information occurred during the hospital stay, especially in the ICU and primarily among relatives. At this stage, the severity of the illness became clear, and relatives described urgent needs for understandable updates and support in situations of decision pressure: “You are the spouse, you have to decide now.” Another added: “You are left completely in the dark.” Several also criticized the lack of clear explanations and written materials: “I would have liked to know what sepsis actually is.”/“You’re not prepared. You don’t know what to expect.”

Survivors described a different pattern. Many said they were unable to process information during the acute phase because they felt cognitively and emotionally overwhelmed in the ICU and early rehabilitation. They began to seek orientation after physical stabilization: “And at some point at home. Then I had a look at all the documents. After the rehab, actually. Yes, exactly when my health was better, when I was a bit stronger, no longer so weak.”

Informational needs remained high after discharge for both groups. Six survivors described a continued need for guidance on long-term effects, rehabilitation, and follow-up care. Two participants (one survivor, one relative) reported ongoing needs well beyond the acute phase. Survivors frequently described receiving little or no information when leaving the hospital. Many did not know what steps to take because no clear guidance had been provided (mentions: 8/12). About half had no scheduled follow-up appointment (mentions: 6/12), and most reported not receiving information on long-term consequences, vaccination recommendations, or continuing care needs (mentions: 10/12). Several summarized this period as an “information vacuum”: “Yes, of course, at first I didn’t know what to do next.”/“No, nothing at all. Nothing was planned at all.”.]

### 3.4. Long-Term Burden on Relatives

Over the longer term, many relatives assumed extensive coordination, caregiving, and emotional responsibilities without feeling adequately informed. One relative described: “My mother can’t do anything anymore. I have to take care of everything. And I still don’t really understand what sepsis actually did to her.” These accounts highlight a sustained informational and emotional burden that persisted well beyond the acute episode. Relatives described intense distress and a sense of being left alone: “I cried in the car every day before I went to the rehabilitation clinic. Every day. I didn’t know how I was going to cope with it all.”/“It was like another world. You were completely left alone.” Insufficient information about the illness and its consequences contributed to prolonged feelings of helplessness and isolation.

### 3.5. Informational Gaps and Post-Discharge Management

**Figure 3:**
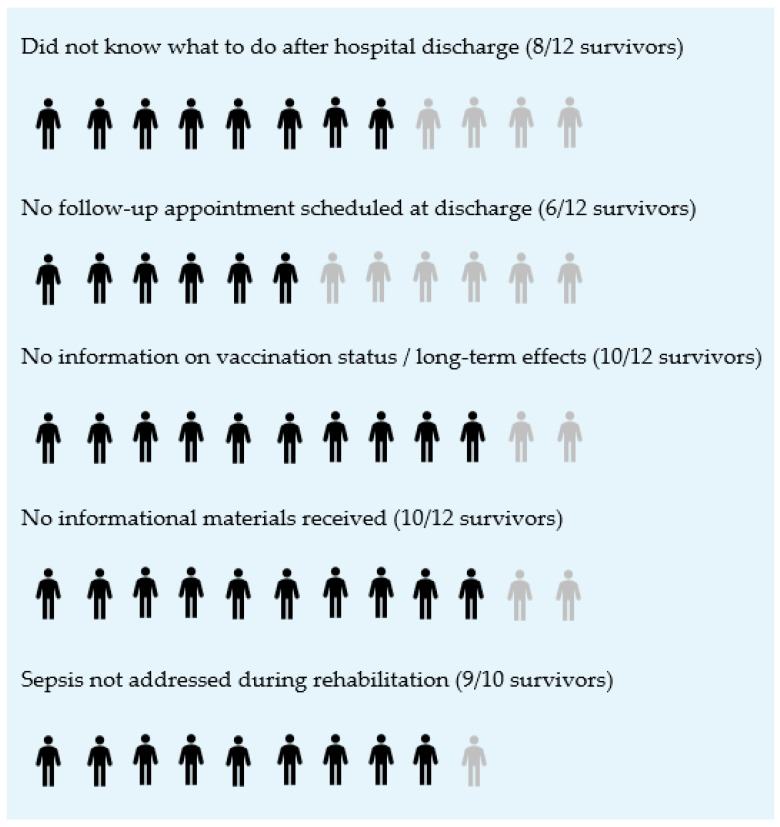
Post-discharge management and informational gaps (Survivors only, *n* = 12).

Many interviewees reported that they had not received any informational materials during their hospital stay (13 of 18; 9 survivors, 4 relatives). As shown in Figure 3, survivors in particular described substantial gaps in post-discharge management and information. These included uncertainty about required care, the absence of scheduled follow-up appointments, limited communication about vaccinations and long-term consequences, a lack of written materials, and no sepsis-specific discussions during rehabilitation.

### 3.6. Preferences for Information Delivery and Content

**Figure 4:**
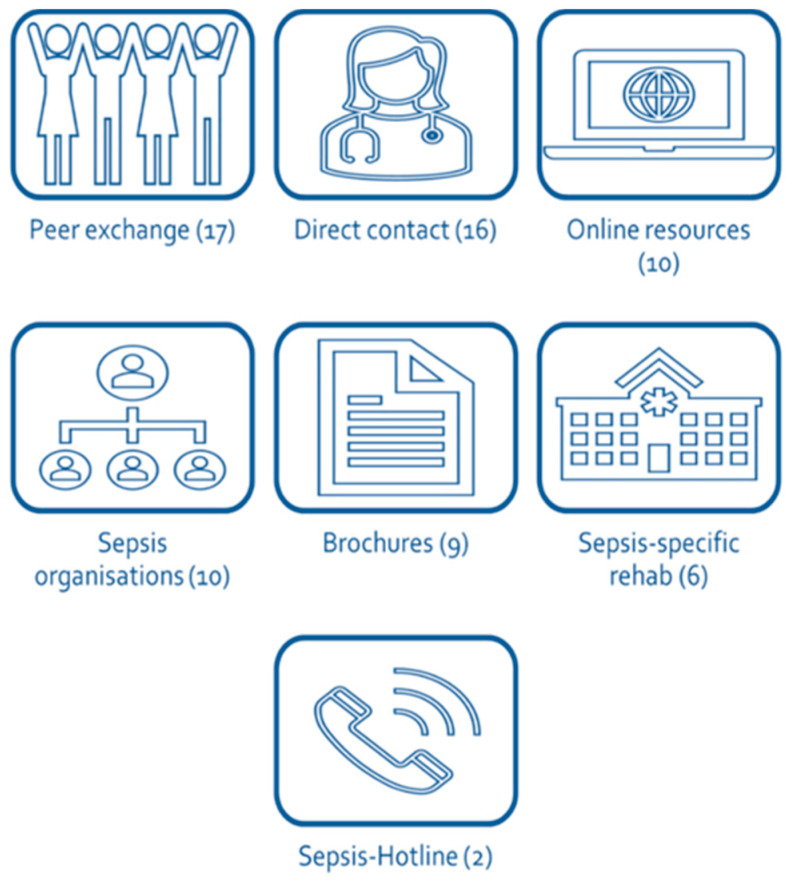
Preferred formats for receiving information (*n* = 18; numbers in brackets indicate mention counts, that is, how often a format was described across all interviews).

Participants expressed consistent preferences across interviews for how information should be delivered. Peer support was the most valued format. Many also emphasized the importance of direct, personal conversations with healthcare professionals. Trusted online resources such as those provided by the Sepsis Foundation and printed materials were viewed as helpful, especially references during recovery. Several interviewees additionally suggested integrating sepsis-focused education into rehabilitation programs and establishing or expanding a dedicated hotline for sepsis-related questions. As shown in Figure 4, these preferences reflect a strong desire for accessible, trustworthy, and personalized information pathways.

**Figure 5:**
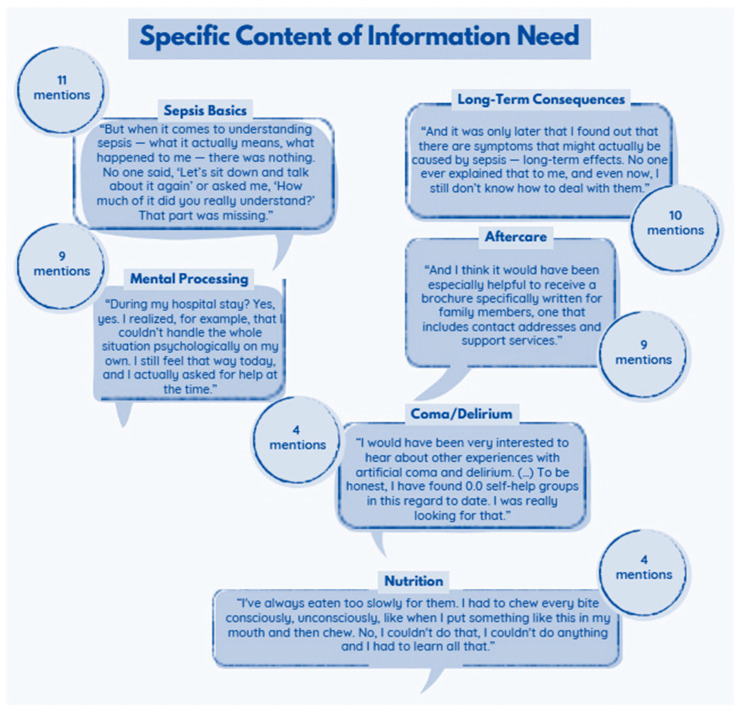
Key content areas of desired sepsis-related information (*n* = 18; number of mentions alongside).

The content participants requested extended well beyond the acute illness. They wanted information about long-term risks, persistent symptoms, and strategies for day-to-day coping. One survivor noted: “And it was only later that I found out that there are symptoms that might actually be caused by sepsis—long-term effects. No one ever explained that to me, and even now, I still don’t know how to deal with them.” Another participant highlighted the absence of basic orientation: “But when it comes to understanding sepsis—what it actually means, what happened to me—there was nothing.” Figure 5 provides an overview of these informational needs.

Participants frequently requested basic knowledge about sepsis: what it is, how it develops, and why it is life-threatening. Many also wanted information about long-term effects, including what to expect after discharge and how symptoms may evolve. A substantial group emphasized psychological consequences and asked for clear explanations of possible emotional and mental health changes. Requests for navigation support were similarly frequent, such as information on whom to contact, how to access services, and where to find peer support. Some participants also sought explanations of coma- or delirium-related cognitive problems and practical advice on nutrition during recovery.

## 4. Discussion

This study describes a phase- and role-specific pattern of informational needs among sepsis survivors and their relatives. During the acute phase, particularly ICU, relatives reported an urgent need for timely, comprehensible, and empathetic updates. Survivors, in contrast, often felt unable to process information during this time and described seeking orientation only after physical and cognitive stabilization. Across care transitions, many participants reported an information vacuum after discharge, including unclear responsibilities, absent or insufficient follow-up-communication, and limited guidance on long-term consequences, vaccinations, or ongoing care. Sepsis-specific information in rehabilitation was also described as rare. Preferred formats included peer support and direct conversations with clinicians, supplemented by trusted online resources and written materials. Desired content ranged from basic explanation of sepsis to practical strategies for coping with long-term physical and psychological sequelae and navigating support services.

Overall, the findings are consistent with previous qualitative studies on sepsis survivorship. Gallop et al. described low awareness of the diagnosis, difficulties accessing appropriate providers, and persistent caregiver burden—patterns that were also reflected in our interviews [14]. Similarly, research on families of critically ill patients highlights acute informational needs, high uncertainty, and the value of repeated and structured communication, while survivors’ own ability to engage with information often re-emerges only after hospital discharge [22,23,24,25]. Evidence from the PICS and post-sepsis literature further documents long-term cognitive, psychological, and physical sequelae including cognitive impairment, anxiety/depression, fatigue, and disability, which helps explain why survivors in our interviews sought orientation on long-term effects and day-to-day coping [4,6,7,8,10,26,27,28].

The gaps described at the transition out of hospital mirror previous findings that ICU discharge summaries often lack the detail required for effective post-ICU care [11]. “Lost in transition” patterns at care interfaces have been observed in other settings as well [29]. Narrative review emphasizes the central role of general practitioners in post-ICU recovery and the importance of structured communication from the hospital to primary care [30]. Similarly to our interviews, studies on rehabilitation highlight inconsistent sepsis-specific content and variable access to aftercare services [10,13,17,31].

Preferences for peer support and for direct communication with clinicians align with evidence that ICU-recovery and peer-support programs can enhance orientation, self-efficacy, and emotional well-being, while clinician encounters can help translate general recommendations into individual guidance [16,24,32].

### 4.1. Potential Implications for Practice

The findings support a phase-specific and role-sensitive approach to information delivery. In the ICU, short and scheduled updates for relatives using clear language and addressing status, uncertainty, and immediate steps may reduce decisional strain [22,23,25]. At discharge, a structured information package that includes follow-up appointments, explanations of long-term effects and vaccination recommendations, and a named contact person could address the most pressing gaps [11,29,30]. During rehabilitation and early recovery, sepsis-specific educational modules (fatigue management, cognitive strategies, mental health, reinfection concerns), peer-support offers, and reliable online or printed resources may match survivors’ delayed readiness and practical needs [13,17,24,32]. Early transfer of concise, action-oriented information to primary care can also support coordinated recovery close to home [30]. These steps frame information as a central component of care quality and correspond to calls for structured survivorship and aftercare services for sepsis survivors [9,10,28].

### 4.2. Why Information Remains Hard to Obtain: Provider Knowledge and System Gaps

Our interviews indicate that informational gaps arise not only at care transitions but also from limited preparedness and confidence among healthcare professionals regarding sepsis-specific knowledge, particularly on long-term sequelae, prevention, and follow-up pathways. A scoping review highlights substantial variation in knowledge and awareness among patients, the general public and healthcare professionals, which complicates consistent counseling [18]. Evidence on information-seeking further indicates that how information is communicated is critical in contexts where time constraints and limited sepsis-specific expertise restrict counseling: a randomized trial demonstrated that text-based and graphical formats differentially influence informed choice [33], and multimodal campaigns offer models for targeted education [34]. Population surveys and digital-trace studies also reveal inconsistent public awareness and fluctuating information demand. In the absence of structured, sepsis-specific follow-up information within the healthcare system, this helps explain why many survivors and relatives rely on general internet sources yet struggle to identify specific and trustworthy information [18,35,36,37]. In the German context, pathway studies describe communication barriers, handover problems, time constraints, and low prioritization of sepsis routines, all of which impede reliable information transfer [38,39]. Non-standardized discharge summaries and weak linkage to primary care leave survivors without a clear point of contact, extending the post-discharge information vacuum reported in our interviews [11,29,30]. Within rehabilitation, programs often focus on generic physical recovery, while sepsis-specific education remains limited [10,13,17]. Addressing these issues may require improved education for healthcare professionals on sepsis survivorship and standardized handover processes that clarify whom to contact, when, and for which concerns—ensuring that information is accessible when patients and families are ready to receive it.

Furthermore, a notable characteristic of our sample is its comparatively young age. This may reflect the recruitment strategy, which relied on digital channels and a university hospital outpatient clinic. These pathways may preferentially engage younger and more health-active survivors. Older survivors, who represent a substantial proportion of the sepsis population and often have high multimorbidity and greater functional limitations, may therefore be underrepresented. This age distribution should be taken into account when interpreting the types of informational needs and preferred formats described here.

Finally, these findings should be interpreted as exploratory and reflect patterns observed within this qualitative sample rather than generalizable conclusions for all sepsis survivors and relatives.

## 5. Limitations

This study has several limitations. First, the sample size is small and the sample is not representative; therefore, the findings cannot be generalized to all sepsis survivors or their relatives. This constraint is inherent to the qualitative, exploratory design, which aims to generate in-depth insights rather than generalizable estimates.

Second, recruitment occurred largely through digital platforms and a university hospital outpatient clinic. This may have introduced a bias toward younger, more digitally literate, and more health-engaged individuals, potentially underrepresenting older survivors, who constitute a substantial proportion of the sepsis population and often experience higher multimorbidity and functional limitations. This demographic pattern may limit the transferability of the findings to older or more clinically complex groups.

Third, the study relied on retrospective self-reporting, which may be affected by recall bias, especially among survivors who experienced delirium or cognitive dysfunction. Survivors with more severe cognitive impairment were likely underrepresented because they may be less able to participate in interviews despite potentially high informational needs. Only limited clinical information was collected (age, ICU treatment, rehabilitation), and no granular clinical variables such as illness severity, organ dysfunction, comorbidities, delirium, mechanical ventilation, or length of ICU stay were available. As a result, findings cannot be contextualized across different clinical trajectories. In addition, we did not systematically collect sociodemographic characteristics such as educational level, employment status, or migration background, which limits the ability to assess variation across socioeconomic or cultural groups.

Fourth, although the study included both survivors and relatives, data saturation was assessed across the dataset as a whole and not separately for these two groups. Given that survivors and relatives may differ substantially in their perspectives, informational needs, and illness experiences, this approach may have obscured group-specific nuances. In a future study, saturation should therefore be assessed separately for survivors and relatives to allow for a more differentiated and methodologically rigorous analysis.

Fifth, we did not systematically distinguish between care settings (ICU, general ward, rehabilitation) in the analysis. Although participants described experiences across these settings, the study was not designed to generate setting-specific comparisons, which limits conclusions about differences in informational needs across environments.

Sixth, triangulation occurred within the research team but did not include multiple data sources, thereby narrowing methodological breadth.

Seventh, as the study was conducted within the German healthcare system, differences in structure and organization in other countries may limit international transferability.

Taken together, these limitations substantially restrict transferability and underscore that the results primarily inform hypothesis generation and future research.

## 6. Conclusions

This study indicates that participants reported gaps in informational support that affect sepsis survivors and their relatives across the illness and recovery trajectory. These gaps—from limited communication during the acute phase to insufficient guidance on long-term follow-up—may hinder recovery and contribute to prolonged physical, cognitive, and emotional challenges. Addressing them requires timely, accessible, and phase-specific information strategies.

Based on the findings, formats that support structured discharge planning, high-quality and tailored educational materials, and opportunities for peer support may help reduce uncertainty and strengthen coping in survivors and families. By mapping how informational needs differ between survivors and relatives and how they evolve across phases of care, the study provides phase- and role-specific insights that can inform the development of future follow-up structures and information tools. Accordingly, the conclusions drawn from this study are intended to inform understanding of informational needs rather than to define their prevalence or distribution across survivor populations.

## Figures and Tables

**Table 1:** Interview guide.

General Information
○ Hello, my name is _______. I’d like to ask you a few questions about your experience with sepsis, or that of your relative.
○ To begin, could you please introduce yourself and tell me your name and gender?
○ When did you or your relative develop sepsis?
○ Were you or your relative treated in an intensive care unit (ICU)?
Hospital Stay
○ Let’s start by talking about your hospital stay.
○ To what extent were you involved in decisions about your medical or nursing care during your time in hospital?
○ Were these decisions discussed with you?
○ Did you feel that the treatments and decisions made about your care were clear and understandable? (Did you understand why each step was taken?)
○ How did you feel about the medical information you received after discharge?
○ Do you think there was room for improvement in that area?
○ *If yes*, what could have been done better?
Discharge and Post-Hospital Care
○ Now, I’d like to talk about the information you received as part of your discharge and follow-up care.
○ Before or during discharge, were there any uncertainties about what to do next?
○ What were your biggest challenges or concerns at that time?
○ Were you able to talk to someone about these concerns?
○ *If yes*, who?
○ Did you receive any materials about follow-up care after sepsis?
○ *If yes*, what kind?
○ Were these materials helpful? Which ones helped more, and which less?
Further Information
○ At what point do you think you would have benefited from more information about sepsis?
○ What were your main concerns at the time of discharge?
○ Were follow-up appointments already scheduled at the time of discharge?
○ *If not*, would you have preferred that?
○ Would you say you knew what steps to take after leaving the hospital?
○ *If not*, what could have helped make things clearer?
Follow-Up Treatment/Rehabilitation
○ After leaving the hospital, did you or your relative receive any follow-up treatment or rehabilitation? (If necessary, please explain what is meant by follow-up care.)
○ *Only for patients who underwent inpatient rehabilitation:*
○ Was sepsis discussed with you during rehab?
○ *If yes*:
○ What exactly was discussed?
○ Did the person providing the information give you any supporting materials?
○ If so, what kind?
○ Which formats do you prefer when receiving information? (e.g., in-person conversations, websites, brochures, etc.)
○ How helpful did you find the materials provided?
○ What additional information would have helped you at that time?
Initial Diagnosis
○ Now I’d like to go back to the moment you first learned about the sepsis diagnosis.
○ Do you remember when this happened?
○ *If yes*:
○ Where and when did you find out?
○ Who informed you that you or your relative had sepsis?
○ How satisfied were you with the explanation given at that time?
○ Were you able to remember the information afterwards?
○ How did you assess your or your relative’s chances of survival?
○ Was the information understandable, or did it contain too many medical terms?
○ How could that conversation have been improved?
○ Were you given any materials when the diagnosis was explained?
○ *If yes*:
○ What kind of materials?
○ How useful were they? (If they were hard to understand, why?)
○ What kind of materials would have helped you understand the situation better (e.g., brochures, websites)?
After Diagnosis: Your Actions and Information Seeking
○ What did you do after learning about the diagnosis?
○ Did you seek out more information on your own?
○ Would it have helped to connect with others affected by sepsis?
Your Knowledge of Sepsis—Then and Now
○ Let’s talk about your knowledge of sepsis.
○ Had you heard of sepsis before?
○ *If yes*, how did you know about it?
○ Were you aware of symptoms that could point to sepsis?
○ *If yes*, how did you learn about them?
○ How would you describe sepsis in your own words?
○ What are the signs that someone might have sepsis?
Prevention and Risk Reduction
○ Do you think it’s possible for you to get sepsis again?
○ Have you received any information about how to reduce the risk of getting sepsis again (e.g., healthy lifestyle, staying up to date on vaccinations)?
○ *If yes*, which suggestions have you followed?
○ What symptoms would alert you to the possibility of sepsis now?
○ Would you like more information about how to recognize sepsis yourself? (yes/no)
Conclusion—Most Urgent Information Needs
○ Finally, I’d like to ask about the time when you most urgently needed information.
○ At what point did you feel the greatest need for information about sepsis?
○ How could that need have been better met?
○ What would have helped you better understand the illness overall?
○ What channels of communication or types of information (e.g., websites, conversations, print materials) would have worked best for you?
Final Remarks
Thank you so much for your time and openness. Do you have any comments, suggestions, or feedback you’d like to share with me or the project team? Is there anything important that we haven’t discussed but you’d still like to mention? You’re also welcome to contact us later if you think of anything else.

Note: If the patient becomes emotionally overwhelmed during the conversation, please refer them to appropriate support services, such as the forum for people affected by sepsis. Italicized content indicates filter questions.

## Data Availability

The original contributions presented in this study are included in the article. Further inquiries can be directed to the corresponding author(s).

## Supplementary Materials

The following supporting information can be downloaded at: https://www.mdpi.com/article/10.3390/jcm15010091/s1, Electronic supplement S1: Definitions of categories and selected original quotes.

## References

[1] Rudd K.E., Johnson S.C., Agesa K.M., Shackelford K.A., Tsoi D., Kievlan D.R., Colombara D.V., Ikuta K.S., Kissoon N., Finfer S. (2020). Global, regional, and national sepsis incidence and mortality, 1990–2017: Analysis for the Global Burden of Disease Study. Lancet.

[2] Fleischmann-Struzek C., Schwarzkopf D., Reinhart K. (2022). Sepsis incidence in Germany and worldwide: Current knowledge and limitations of research using health claims data. Med. Klin. Intensivmed. Notfmed..

[3] Fleischmann-Struzek C., Ditscheid B., Rose N., Spoden M., Wedekind L., Schlattmann P., Günster C., Reinhart K., Hartog C.S., Freytag A. (2023). Return to work after sepsis—A German population-based health claims study. Front. Med..

[4] Iwashyna T.J., Ely E.W., Smith D.M., Langa K.M. (2010). Long-term Cognitive Impairment and Functional Disability Among Survivors of Severe Sepsis. JAMA.

[5] Sell S., Fleischmann-Struzek C., Spoden M., Rosendahl J. (2025). Mental health in the first year after ICU-treated sepsis: Analysis of administrative diagnoses in German health claims data. Gen. Hosp. Psychiatry.

[6] Gupta L., Subair M.N., Munjal J., Singh B., Bansal V., Gupta V., Jain R. (2024). Beyond survival: Understanding post-intensive care syndrome. Acute Crit. Care.

[7] Inoue S., Nakanishi N., Sugiyama J., Moriyama N., Miyazaki Y., Sugimoto T., Fujinami Y., Ono Y., Kotani J. (2022). Prevalence and Long-Term Prognosis of Post-Intensive Care Syndrome after Sepsis: A Single-Center Prospective Observational Study. J. Clin. Med..

[8] Prescott H.C., Angus D.C. (2018). Enhancing Recovery From Sepsis: A Review. JAMA.

[9] Elliott D., Davidson J.E., Harvey M.A., Bemis-Dougherty A., Hopkins R.O., Iwashyna T.J., Wagner J., Weinert C., Wunsch H., Bienvenu O.J. (2014). Exploring the scope of post-intensive care syndrome therapy and care: Engagement of non-critical care providers and survivors in a second stakeholders meeting. Crit. Care Med..

[10] Rousseau A.F., Prescott H.C., Brett S.J., Weiss B., Azoulay E., Creteur J., Latronico N., Hough C.L., Weber-Carstens S., Vincent J.L. (2021). Long-term outcomes after critical illness: Recent insights. Crit. Care.

[11] Hauschildt K.E., Hechtman R.K., Prescott H.C., Iwashyna T.J. (2022). Hospital Discharge Summaries Are Insufficient Following ICU Stays: A Qualitative Study. Crit. Care Explor..

[12] Huang C.Y., Daniels R., Lembo A., Hartog C., O’Brien J., Heymann T., Reinhart K., Nguyen H.B., Sepsis Survivors Engagement Project (SSEP) (2019). Life after sepsis: An international survey of survivors to understand the post-sepsis syndrome. Int. J. Qual. Health Care.

[13] Winkler D., Rose N., Freytag A., Sauter W., Spoden M., Schettler A., Wedekind L., Storch J., Ditscheid B., Schlattmann P. (2023). The Effects of Postacute Rehabilitation on Mortality, Chronic Care Dependency, Health Care Use, and Costs in Sepsis Survivors. Ann. Am. Thorac. Soc..

[14] Gallop K.H., Kerr C.E.P., Nixon A., Verdian L., Barney J.B., Beale R.J. (2015). A Qualitative Investigation of Patients’ and Caregivers’ Experiences of Severe Sepsis. Crit. Care Med..

[15] Born S., Matthäus-Krämer C., Bichmann A., Boltz H.-S., Esch M., Heydt L., Sell S., Streich K., Scherag A., Reinhart K. (2023). Sepsis survivors and caregivers perspectives on post–acute rehabilitation and aftercare in the first year after sepsis in Germany. Front. Med..

[16] Sevin C.M., Boehm L.M., Hibbert E., Bastin A.J., Jackson J.C., Meyer J., Quasim T., Bakhru R.N., Montgomery-Yates A., Slack A. (2021). Optimizing Critical Illness Recovery: Perspectives and Solutions from the Caregivers of ICU Survivors. Crit. Care Explor..

[17] Smith-Turchyn J., Alborzi M., Hong J., Hvizd J.L., McKenney S., Newman A.N.L., Rochwerg B., Kho M.E. (2025). Rehabilitation needs, preferences, barriers, and facilitators of individuals with sepsis: A qualitative study. Int. J. Nurs. Stud. Adv..

[18] Fiest K.M., Krewulak K.D., Brundin-Mather R., Leia M.P., Fox-Robichaud A., Lamontagne F., Leigh J.P., For Sepsis Canada (2022). Patient, Public, and Healthcare Professionals’ Sepsis Awareness, Knowledge, and Information Seeking Behaviors: A Scoping Review. Crit. Care Med..

[19] Fleischmann-Struzek C., Rose N., Ditscheid B., Draeger L., Dröge P., Freytag A., Goldhahn L., Kannengießer L., Kimmig A., Matthäus-Krämer C. (2024). Understanding health care pathways of patients with sepsis: Protocol of a mixed-methods analysis of health care utilization, experiences, and needs of patients with and after sepsis. BMC Health Serv. Res..

[20] Mayring P., Mey G., Mruck K. (2010). Qualitative Inhaltsanalyse. Handbuch Qualitative Forschung in der Psychologie.

[21] Tong A., Sainsbury P., Craig J. (2007). Consolidated criteria for reporting qualitative research (COREQ): A 32-item checklist for interviews and focus groups. Int. J. Qual. Health Care.

[22] Czerwonka A.I., Herridge M.S., Chan L., Chu L.M., Matte A., Cameron J.I. (2015). Changing support needs of survivors of complex critical illness and their family caregivers across the care continuum: A qualitative pilot study of Towards RECOVER. J. Crit. Care.

[23] King J., O’Neill B., Ramsay P., Linden M.A., Darweish Medniuk A., Outtrim J., Blackwood B. (2019). Identifying patients’ support needs following critical illness: A scoping review of the qualitative literature. Crit. Care.

[24] McPeake J., Boehm L.M., Hibbert E., Bakhru R.N., Bastin A.J., Butcher B.W., Eaton T.L., Harris W., Hope A.A., Jackson J. (2020). Key Components of ICU Recovery Programs: What Did Patients Report Provided Benefit?. Crit. Care Explor..

[25] Stayt L.C., Venes T.J. (2019). Outcomes and experiences of relatives of patients discharged home after critical illness: A systematic integrative review. Nurs. Crit. Care.

[26] Hodgson C.L., Udy A.A., Bailey M., Barrett J., Bellomo R., Bucknall T., Gabbe B.J., Higgins A.M., Iwashyna T.J., Hunt-Smith J. (2017). The impact of disability in survivors of critical illness. Intensive Care Med..

[27] Needham D.M., Davidson J., Cohen H., Hopkins R.O., Weinert C., Wunsch H., Zawistowski C., Bemis-Dougherty A., Berney S.C., Bienvenu O.J. (2012). Improving long-term outcomes after discharge from intensive care unit: Report from a stakeholders’ conference. Crit. Care Med..

[28] Prescott H.C., Iwashyna T.J., Blackwood B., Calandra T., Chlan L.L., Choong K., Connolly B., Dark P., Ferrucci L., Finfer S. (2019). Understanding and Enhancing Sepsis Survivorship. Priorities for Research and Practice. Am. J. Respir. Crit. Care Med..

[29] Gadbois E.A., Tyler D.A., Shield R., McHugh J., Winblad U., Teno J.M., Mor V. (2019). Lost in Transition: A Qualitative Study of Patients Discharged from Hospital to Skilled Nursing Facility. J. Gen. Intern. Med..

[30] Vrettou C.S., Mantelou A.G. (2025). Supporting Post-ICU Recovery: A Narrative Review for General Practitioners. Diseases.

[31] van der Slikke E.C., Beumeler L.F.E., Holmqvist M., Linder A., Mankowski R.T., Bouma H.R. (2023). Understanding Post-Sepsis Syndrome: How Can. Clinicians Help?. Infect. Drug Resist..

[32] Clarke R., Chow H., Kerrison K. (2023). An Intensive Care Unit peer support group: Participants’ views on format, content and the impact on recovery journeys. J. Intensive Care Soc..

[33] Debbeler L.J., Pohrt A., Fleischmann-Struzek C., Schwarzkopf D., Born S., Reinhart K., Wegwarth O. (2022). Text-Based vs. Graphical Information Formats in Sepsis Prevention and Early Detection: A Randomized Controlled Trial on Informed Choice. J. Clin. Med..

[34] Abels W., Reinhart K., Neugebauer E., Wulkotte E., Toubekis E., Piedmont S., Born S., Rieck T., Wegwarth O., Spies C. (2024). Improving prevention and early detection of sepsis among patient groups at risk: Introducing a model for a multimodal information campaign—The SepWiss study protocol. PLoS ONE.

[35] Jabaley C.S., Blum J.M., Groff R.F., O’Reilly-Shah V.N. (2018). Global trends in the awareness of sepsis: Insights from search engine data between 2012 and 2017. Crit Care.

[36] Jabaley C.S., Groff R.F., Barnes T.J., Caridi-Scheible M.E., Blum J.M., O’Reilly-Shah V.N. (2019). Sepsis information-seeking behaviors via Wikipedia between 2015 and 2018: A mixed methods retrospective observational study. PLoS ONE.

[37] Parsons Leigh J., Brundin-Mather R., Moss S.J., Nickel A., Parolini A., Walsh D., Bigham B.L., Carter A.J.E., Fox-Robichaud A., Fiest K.M. (2022). Public awareness and knowledge of sepsis: A cross-sectional survey of adults in Canada. Crit. Care.

[38] Draeger L., Fleischmann-Struzek C., Bleidorn J., Kannengiesser L., Schmidt K., Apfelbacher C., Matthaeus-Kraemer C. (2025). Healthcare Professionals’ Perspectives on Sepsis Care Pathways—Qualitative Pilot Expert Interviews. J. Clin. Med..

[39] Matthaeus-Kraemer C.T., Thomas-Rueddel D.O., Schwarzkopf D., Rueddel H., Poidinger B., Reinhart K., Bloos F. (2016). Crossing the handover chasm: Clinicians’ perceptions of barriers to the early detection and timely management of severe sepsis and septic shock. J. Crit. Care.

