# Supplementary material for: “Lost in Transition”: Informational Needs of Sepsis Survivors and Their Relatives Across the Care Trajectory—A Qualitative Study
Source: J Clin Med. 2025 Dec 23;15(1):91. doi: 10.3390/jcm15010091 (PMC12787246; doi:10.3390/jcm15010091)
Supplement: Supplementary file 1 [file jcm-15-00091-s001.zip › jcm-4030691-supplementary.pdf]

| List of codes | Definition | Citation | Number of mentions |
|---|---|---|---|
| InfoNeeds | Participants are asked about the specific content of their information needs | | |
| SepsisBasics | Relatives or patients stated they would have preferred more information about medical basics of sepsis | "But when it comes to understanding sepsis — what it actually means, what happened to me — there was nothing. No one said, ‘Let’s sit down and talk about it again’ or asked me, ‘How much of it did you really understand?’ That part was missing.” | 11 |
| LongTerm | Relatives or patients stated they would have preferred more information about long term consequences of sepsis | "And it was only later that I found out that there are symptoms that might actually be caused by sepsis — long-term effects. No one ever explained that to me, and even now, I still don’t know how to deal with them." | 10 |
| Aftercare | Relatives or patients stated they would have preferred more information about contact points for aftercare | "And I think it would have been especially helpful to receive a brochure specifically written for family members, one that includes contact addresses and support services. | 9 |
| MentalProc | Relatives or patients stated they would have preferred more information about mental processing of sepsis | "During my hospital stay? Yes, yes. I realized, for example, that I couldn’t handle the whole situation psychologically on my own. I still feel that way today, and I actually asked for help at the time." | 9 |
| ComaDelir | Here, relatives or patients said they would have liked to learn more about coma and delir | "I would have been very interested to hear about other experiences with artificial coma and delirium. (...) To be honest, I have found o.o self-help groups in this regard to date. I was really looking for that" | 4 |
| Nutrition | Relatives or patients stated they would have preferred more information about nutrition | "I've always eaten too slowly for them. I had to chew every bite consciously, unconsciously, like when I put something like this in my mouth and then chew. No, I couldn't do that, I couldn't do anything and I had to learn all that." | 4 |
| InfoNeedTime | The point in time at which the need for information was highest is assessed. | | |
| Time_ICU | Time of greatest need for information: during ICU stay | "So when he was still in intensive care." | 6 |
| Time_Post | The time of greatest need for information was post-stationary | "And at some point at home. Then I had a look at all the documents. After the rehab, actually. Yes, exactly when my health was better, when I was a bit stronger, no longer so weak." | 6 |
| Time_Inpt | The time of greatest need for information was during an inpatient stay in a hospital or rehabilitation centre | "Yes, of course, right at the hospital" | 5 |
| Time_Cont | There was a continuous need for information | "So actually on all of them." | 2 |

| | | | |
|---|---|---|---|
| InfoSources | Which are the preferred sources of information for the patient or relatives? | | |
| SelfHelp/ Peer Exchange | The interviewees' preferred source of information is the self-help group or other affected people | "And then I found a self-help group near where I live. That's about 30 or 40 kilometers. I only received an email and an answer yesterday. So now I want to see if I can somehow find a place where this support group is, to see what people say who have experienced the same thing or something similar." | 17 |
| DirectContact | Direct personal contact is named as the preferred source of information | "I would have liked to have had that in a personal conversation, because there are also questions then and yes, yes, that's it, I don't think it's unimportant either" | 16 |
| SepsisOrgs | The preferred, or one of the preferred, sources of information is the Sepsisstiftung, Deutsch Sepsis-Hilfe or Sepsis Dialog | "I said yes, because I then had the Sepsis-Hilfe and I knew if something happened, you could call them, and they would be there for you 24/7” | 10 |
| Internet | The Internet is named as the preferred source of information. | "Via the internet and read up on it" | 10 |
| Brochures | Brochures were named as the preferred source of information. | "For example, simply on an information sheet that doesn't overwhelm them, that can be taken along.” | 9 |
| SpecRehab | The interviewees' preferred source of information is a stay at a specific rehab Centre | "Not so much is done. For Covid, long Covid there are so many rehabs. There are now. There's nothing for sepsis." | 6 |
| Hotline | The preferred, or one of the preferred sources of information, is a hotline | "Yes, yes, at least whether there was any written information material or I was somehow given a number. The one that tells me you can call if you have any questions, because I mean, it's all limited in the clinic, that's true. But the fact that you can simply call somewhere if you need help or if you have questions or whatever, that's reassuring." | 2 |
| Question of more information/media presence | Patient or relative is asked whether he/she would like more information and/or media presence about sepsis | | |
| More information/media presence desired | Patients or relatives would like more information or media presence | "And that's where you have to start. And I've realized that if we start with the medical staff, we've lost. We must start at grassroots level. The population must exert this pressure on medicine, on doctors in outpatient clinics, on doctors in hospitals, because only an educated population is also a responsible population. They can then say I want it that way too." | 17 |

| | | | |
|---|---|---|---|
| Interviewee considers him/herself sufficiently informed | The interviewee considers themselves to have sufficient information on sepsis | "I'm enlightened, but as I said, I'd say I've read about 90 per cent of it." | 2 |
| Information on vaccination/nutrition | This section concerns vaccination and nutrition | | |
| No information was given on vaccination/nutrition | Patients or relatives were not explicitly informed about special vaccination or nutrition | "No, I haven't been told or explained that yet." | 11 |
| Advice was given on vaccination/nutrition | Patient or relative was explicitly advised of special vaccination or special nutrition after surviving the illness | "We have good vaccination protection. So, our GP recommended the pneumococcal vaccination, which we both had. We'd already had the flu vaccination and we also had the tetanus vaccination. Our immunization status was fine before. We just hadn't had the pneumococcal vaccination yet. And when my mum had it, she's now 96, but we're now completely behind the times and everything is always covered ok." | 4 |
| Patients have not complied with instructions | The instructions on special vaccination and special nutrition after surviving sepsis were not followed | "No, I'm not going to do that either" | 1 |
| Patient has complied with instructions on nutrition/vaccination protection | The instructions on special vaccination protection and special nutrition after surviving sepsis were followed | | 0 |
| Provision of information material Hospital | The question regarding the provision of informational material in hospital. | | |
| There was no information material at any time | At no time did patients or relatives receive information material from the hospital, rehab centre or outpatient doctors. | "There was no such thing" | 12 |
| There were contact people for non-medical questions during hospital stay | Patients / relatives had contact persons for non-medical matters during their hospitalization | “There was a lady in charge, Mrs. L., who takes care of patients after their hospital stay. Oh dear, she helped me so much. She was the only one who gave me advice. You get letters, and you don't know where to start. Then there's the care, the health insurance. What do we do? Everyone wants something from you, and you have no idea.” | 7 |
| No information material in the hospital | No information material was provided in the hospital. | "No, nothing at all. I was still surprised, but it only occurred to me later, much later, after I had informed myself about sepsis." | 7 |

| | | | |
|---|---|---|---|
| Time of the first diagnosis notification | The point in time when the interviewee was first confronted with the diagnosis of sepsis | | |
| Time_ICU | The first diagnosis notification was on ICU | " It was at the hospital when I wanted to visit my husband in intensive care." | 10 |
| Pre-inpatient (emergency room, family doctor) | The first diagnosis notification was before the inpatient stay | "Yes, exactly" | 2 |
| Time_Post | The first diagnosis notification was post-inpatient | | 0 |
| Loss of information in medical dialogue | Assessment of communication with medical staff | | |
| Misunderstanding due to emotions and/or cognitive limitations | Enlightenment colored by emotions. | "Because you're in a state of shock at first. I also realize from your questions that you can't take it in. I think to myself, I don't even know anymore. That was so blatant." | 7 |
| Language barriers due to specialized vocabulary | Were there any comprehension problems due to the use of specialized language? | "I said I don't understand your communication. You must talk to me in a way that I can understand. And then he didn't understand, and I said I'm not an intensive care doctor, I'm not a senior doctor, how am I supposed to understand that?" | 6 |
| Medical information was easy to understand | The dialogue with doctors was understandable even as a medical layperson. | "That was understandable. It was expressed in a way that I understood. I'm already talking about urosepsis and cystitis and things like that. If you deal with it, then you can do that. But I actually understood all of that." | 4 |
| Information source after diagnosis | Preferred sources of information | | |
| Internet | The interviewees' preferred source of information after diagnosis was the Internet | "Somehow started researching one thing or another on my mobile phone" | 16 |
| Outpatient doctors | Outpatient doctors were named as the or one of the preferred sources of information | "Yes, from my neurologist, who is still looking after me. She really explained it to me first, so that I understood what had happened." | 7 |
| Sepsis-Hilfe/Sepsis-Stiftung/Sepsis-Dialog/WSD | Preferred source of information was Sepsis-Hilfe/Sepsis-Stiftung/Sepsis-Dialog/WSD | "And I then came across the Sepsis-Stiftung relatively quickly." | 6 |

| | | | |
|---|---|---|---|
| Relatives/acquaintances | Relatives/acquaintances were named as the or one of the preferred sources of information | "Okay, but I can tell you from top to bottom that if it hadn't been for my one son, I wouldn't even know what I had today, a year and a half later" | 6 |
| Diagnosis | Circumstances of the diagnosis sepsis | | |
| Doctor | The first person to diagnose sepsis was a doctor | "A doctor has already done that." | 10 |
| Relatives | Relatives were the first to report the diagnosis of sepsis | "After my son explained to me what was wrong with me, I understood everything." | 4 |
| unknown | The circumstances of the announcement of the diagnosis are unknown or not remembered | “I don't remember. My doctor or my husband? I don't remember exactly. Unfortunately.” | 2 |
| own research | Patients or relatives have made the diagnosis of sepsis themselves through their own research | "I always told the first people I spoke to again after the induced coma that I had pneumonia. And it's true, it was there too, but the actual thing that went really wrong and why I had a tube down my throat was the septicemia and it was only through my own research" | 2 |
| noticed in symptoms | person presumes sepsis based on the symptoms | | 0 |
| Sepsis knowledge | This section concerns the knowledge of relatives/patients about the disease. | | |
| Lack of knowledge among medical staff | Medical staff are perceived as having little or no expertise in relation to sepsis. | "To come back to what really shocked me, after what I know now, I realized how little the doctors know." | 8 |
| Probability of re-sepsis | Interviewees are asked about re-sepsis. | | |
| Affected person considers re-sepsis possible | Affected person considers re-sepsis possible | “Yes, I believe that one is not immune to that now.” | 12 |
| Considers re-sepsis rather unlikely | Affected person considers re-sepsis rather unlikely | "And I think I'll be spared that, that I won't get septicemia again." | 1 |
| had re-sepsis | Patient had a re-sepsis | “Well, I would say yes, yes. I left the service in 2019. Did I have to go to the hospital in an ambulance. And there I had another case of early sepsis. A year later” | 1 |
| Call Definition Sepsis | Patient or relative is asked to give a definition of sepsis in their own words | | |
| Definition of sepsis in your own words | The interviewee should give the definition of sepsis in their own words | "Infection of the body that gets out of control." | 13 |
| correct | The interviewee can correctly define sepsis. | "Erm, well, I can try. As I understand it, it's actually the most serious, largest internal infection that enters the bloodstream and spreads throughout the body." | 12 |

| | | | |
|---|---|---|---|
| Sepsis knowledge before / now | The person's prior knowledge about the illness | | |
| Sepsis knowledge before | Patient or relative shares the knowledge they had about sepsis before desease | "Honestly, not at all about the painting itself. If I'd had the knowledge back then that I have today, it wouldn't have got this far." | 13 |
| Blood poisoning | The interviewee states that septicemia is a synonym for blood poisoning. | "So actually, that's all I knew. Sepsis, the term for blood poisoning." | 10 |
| red line | The interviewee states that a red line can be found on the body in the case of sepsis. | “Yes, you're supposed to know that you prick yourself on a rusty nail. And then you somehow see a red line on the vein.” | 7 |
| Alarm sign sepsis/sensitization | Reported symptoms of sepsis | | |
| Fever/chills | The interviewee reports fever and chills as symptoms of sepsis | "Trembling, fever" | 17 |
| Extreme feeling of illness/poor general condition | The interviewee reports feeling extremely ill/poor gerneral consition as a symptom of sepsis | "So, this extremely poor general condition" | 11 |
| CNS | The interviewee reports CNS disorders as a symptom of sepsis | "apathetic, barely responsive" | 10 |
| Signs of a cold/infection | The interviewee reports signs of a cold/infection as a symptom of sepsis | "Like getting the flu when you have a cold" | 6 |
| Pain | The interviewee reports pain as a symptom of sepsis | "Back pain, he came to hospital with back pain" | 5 |
| Shortness of breath/tachypnoea | The interviewee reports tachypnoea as a symptom of sepsis | "rapid breathing" | 5 |
| Vomiting/nausea/loss of appetite | The interviewee reports vomiting/nausea/loss of appetite as a symptom of sepsis | "There was a lot of nausea." | 4 |
| Festering wounds | The interviewee reports suppurating wounds as a symptom of sepsis | "Wound and then he gets some germs in it" | 4 |
| Low blood pressure/dizziness | The interviewee reports low blood pressure/dizziness as a symptom of sepsis | "low blood pressure and yes, dizziness" | 3 |
| High pulse | A rapid pulse is reported as a symptom of sepsis | "fast heartbeat" | 2 |

| | | | |
|---|---|---|---|
| marbled/pale skin | Marbled/pale skin is reported as a symptom of sepsis | "marbled skin" | 2 |
| Cystitis | The interviewee reports bladder inflammation as a symptom of sepsis | "And I pay extreme attention to my body. How high the load is. And I got a bladder infection again last week for the first time, so I'm vigilant. Go to the doctor straight away, take antibiotics and stuff" | 1 |
| Provision of materials during rehab | This section concerns the provision of materials during rehab | | |
| There was no information material during rehab provided | The rehab provider did not provide any information material on sepsis. | "From them? Nothing at all?" | 3 |
| During rehab there were materials provided | Materials were provided during rehab. | | 0 |
| Evaluation Communication during rehab also criticism | Review of communication during rehab | | |
| Sepsis/consequences were not discussed during rehab | Sepsis and its consequences were not discussed during rehab | “Rehab wasn't that helpful either. Rehab was extremely helpful, I have to say. But in terms of understanding sepsis, what does that mean? And we'll talk to you about it again and explain what it was. Or ask you how much you understood. That did not happen.” | 10 |
| Sepsis/consequences were discussed during rehab | The clinical picture of sepsis and its consequences were discussed during rehab. | “She explained it to me again. That's right. But in rehab, which was right after the hospital, after the inpatient treatment. That's where understanding began.” | 2 |
| AA-General | General information | | |
| ICU stay | The question of whether or not an ICU stay occurred | | |
| Yes | ICU stay has taken place. | | 18 |
| no | no ICU | | 0 |
| Rehabilitation stay | The question of whether a rehabilitation stay occured | | |
| **yes** | The rehabilitation stay has taken place | | 14 |
| **no** | The rehabilitation stay has not  taken place | | 2 |
| **Not yet** | The rehabilitation stay has not yet taken place | | 2 |
| **Master data** | | | |
| Patient | Interviewee is a patient | | 12 |
| Relative | The interviewee is a relative. | | 6 |

| | | | |
|---|---|---|---|
| School-leaving certificate | What school-leaving qualification does the interviewee have? | | 8 |
| Gainful employment | Information is provided here on whether the interviewee is employed at the time of the interview. | | 10 |
| Gender | What gender is the interviewee? | | 10 |
| Age of the interviewee | How old is the interviewee? | | 15 |
| Population of the place of the interviewee | How many inhabitants does the place of residence of the interviewee have? | | 16 |
| Distance to the nearest hospital | How far (km) is the interviewee's place of residence from the nearest hospital? | | 17 |
| AB supply area: ICU | This section concerns the traceability of decisions on the ICU, communication with medical and non-medical staff on the ICU, miscommunication on the ICU and integration on the ICU. | | 0 |
| Transparency of decisions on ICU | The issue of the transparency of medical decisions | | |
| ICU decisions were not transparent | Decisions and measures could not be understood in terms of meaning | "That? No, I don't think I understood it. Sure, because my head wasn't good either." | 7 |
| ICU decisions were transparent | Decisions and measures could be understood in terms of their meaning | "It was all comprehensible to me." | 7 |
| Evaluation of communication to nursing on ICU | Evaluation of communication with nursing staff on the ICU | | |
| Positive communication with nursing staff on ICU | Communication with nursing staff on the ICU is reported positively | "That really was a ten for me. I was a very extreme special case. But so were they. Even the care situation is great." | 9 |
| Negative communication with nursing staff on ICU | Negative reports on communication with nursing staff on the ICU | "At HSK nurses, I would say it was a five." | 3 |
| Question Communication doctors ICU | Question about communication with the doctors on the ICU | | |

| | | | |
|---|---|---|---|
| Communication/flow of information with doctors is praised | Examples of satisfactory communication with doctors | "Always explained everything to me very nicely" | 12 |
| Negative assessment Communication doctors ICU | Negative assessment of communication on ICU | "All in all, yes, except for the flow of information. What's wrong with it? What are the dangers, where does it come from? So none of us got a precise explanation." | 5 |
| Miscommunication on ICU | Communicative misunderstandings on ICU. | | |
| Significance of the disease not communicated | Examples are given here of when the significance of sepsis was not communicated | "Yes, well, sepsis is somehow a bigger thing or something, nobody really explained that to me." | 6 |
| Lack of empathy by the staff | Examples of a lack of empathy on the part of ICU staff are cited here | "A bit of empathy would have been useful, so unfortunately it takes time and they just don't have that. And I think a little empathy would have been useful." | 5 |
| Inadequate patient communication with reduced responsiveness | Examples of inadequate patient communication with reduced responsiveness | “But I still didn't know what was going on, and no one talked to me about it or even tried to.” | 3 |
| Relatives felt disruptive | This section concerns examples where relatives have felt disruptive on ICU | “Yes, there was a doctor who frightened me. [...] And you could tell that he was incredibly condescending, and yes, he signaled that too. Yes, and now I'm done and want to go back and not ask any more questions.” | 1 |
| Integration on ICU | Involvement in the ICU. | | |
| Integration into ICU has taken place | Patient or relatives were involved in the ICU and informed about the purpose of measures | "I then moved on relatively quickly to Hannover at the MHH, where I was clear enough in my head to be involved in the decisions." | 8 |
| Could not take place | Integration could not take place | "I didn't realize any of this because I was in an induced coma the whole time." | 6 |
| Relatives: Integration on ICU did not take place | Patient or relatives were not involved in ICU | “I specifically informed them that I would be available day and night if anything happened, and I drove to the hospital. It was a 15-minute drive, so not far. Fifteen minutes is manageable, so they could have informed me. However, for some reason, I was not permitted to do so. Presumably, this was to avoid overwhelming me, I suppose.” | 2 |
| Behavior after discharge from hospital | Behavior during the discharge procedure is assessed. | | |

| | | | |
|---|---|---|---|
| Patients did not know what to do after discharge | Patients/relatives did not know what to do after being discharged from hospital | "Yes, of course, at first I didn't know what to do next." | 12 |
| Patients/relatives knew what to do after discharge | Patients and relatives knew what to do after being discharged from hospital | “Yes. Yes, I knew that.” | 4 |
| Follow-up examinations | The question is whether follow-up examinations were planned during discharge. | | |
| No follow-up examinations were planned during discharge | At the time of discharge, no follow-up visits to outpatient or inpatient doctors were planned | "No, nothing at all. Nothing was planned at all." | 9 |
| Follow-up examinations were already planned during discharge | At the time of discharge from hospital, follow-up examinations were already planned with the family doctor or specialist, or follow-ups in hospital | "Follow-up examination from hospital but not a regular doctor's appointment" | 7 |
